# Poly[[(μ_4_-1,3,5-triamino-1,3,5-tride­oxy-*cis*-inositol)sodium] bromide]

**DOI:** 10.1107/S1600536813005618

**Published:** 2013-03-02

**Authors:** Guido J. Reiss, Kaspar Hegetschweiler

**Affiliations:** a Institut für Anorganische Chemie und Strukturchemie, Lehrstuhl II: Material- und Strukturforschung, Heinrich-Heine-Universität Düsseldorf, Universitätsstrasse 1, D-40225 Düsseldorf, Germany; b Fachrichtung Chemie, Universität des Saarlandes, Postfach 151150, D-66041 Saarbrücken, Germany

## Abstract

In the structure of the title compound, {[Na(C_6_H_15_N_3_O_3_)]Br}_*n*_, the sodium cation and the bromide anion are both located on threefold rotation axes. The sodium cation is bonded to the three hy­droxy groups of one 1,3,5-triamino-1,3,5-tride­oxy-*cis*-inositol (taci) ligand, with the taci ligand residing around the same threefold rotation axis as the sodium ion. The coordination sphere of the sodium ion is completed by three amino groups of three neighbouring taci mol­ecules. Hence, this type of coordination constitutes a three-dimensional open framework with channels along the *c* axis which are filled with the bromide counter-anions. Each bromide ion forms three symmetry-related hydrogen bonds to both the hy­droxy and the amino groups of neighbouring taci ligands.

## Related literature

The crystal structure of an Na–bis-taci complex has been reported by Bartholomä *et al.* (2010 ▶). Puckering parameters were calculated according to Cremer & Pople (1975 ▶). For a preliminary preparation and characterization of the title compound, see: Egli (1994 ▶). For a general overview of the coordination chemistry of taci, see: Hegetschweiler (1999 ▶). The crystal structure of a Cu^II^–taci complex has been reported by Reiss *et al.* (1998 ▶). For the crystal structure of a monoprotonated taci salt, see: Reiss *et al.* (1999 ▶).
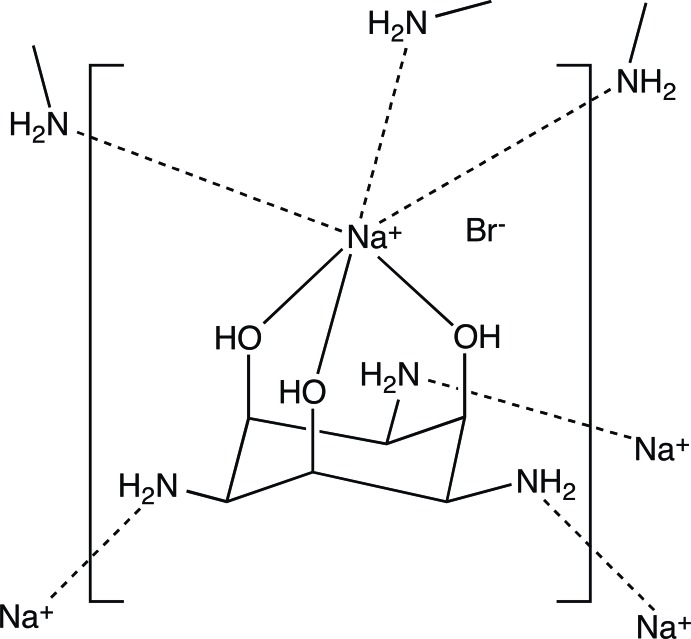


## Experimental

### 

#### Crystal data

[Na(C_6_H_15_N_3_O_3_)]Br
*M* *_r_* = 280.10
Trigonal, 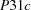

*a* = 8.0491 (10) Å
*c* = 8.8953 (18) Å
*V* = 499.10 (13) Å^3^
*Z* = 2
Mo *K*α radiation
μ = 4.15 mm^−1^
*T* = 153 K
0.57 × 0.45 × 0.28 mm

#### Data collection

Siemens P4 diffractometer
Absorption correction: integration (*XPREP*; Bruker, 2008 ▶) using indexed faces *T*_min_ = 0.101, *T*_max_ = 0.331
3954 measured reflections
690 independent reflections
682 reflections with *I* > 2σ(*I*)
*R*_int_ = 0.077
3 standard reflections every 100 reflections intensity decay: none

#### Refinement

*R*[*F* ^2^ > 2σ(*F* ^2^)] = 0.036
*wR*(*F* ^2^) = 0.078
*S* = 1.05
690 reflections
59 parameters
3 restraints
H atoms treated by a mixture of independent and constrained refinement
Δρ_max_ = 0.42 e Å^−3^
Δρ_min_ = −0.55 e Å^−3^
Absolute structure: Flack (1983 ▶), 340 Friedel pairs
Flack parameter: −0.03 (3)

### 

Data collection: *XSCANS* (Siemens, 1994 ▶); cell refinement: *XSCANS*; data reduction: *XSCANS* and *XPREP* (Bruker, 2008 ▶); program(s) used to solve structure: *SHELXS97* (Sheldrick, 2008 ▶); program(s) used to refine structure: *SHELXL97* (Sheldrick, 2008 ▶); molecular graphics: *DIAMOND* (Brandenburg, 2012 ▶); software used to prepare material for publication: *SHELXL97* and *PLATON* (Spek, 2009 ▶).

## Supplementary Material

Crystal structure: contains datablock(s) global, I. DOI: 10.1107/S1600536813005618/wm2727sup1.cif

Structure factors: contains datablock(s) I. DOI: 10.1107/S1600536813005618/wm2727Isup2.hkl

Additional supplementary materials:  crystallographic information; 3D view; checkCIF report

## Figures and Tables

**Table 1:** Hydrogen-bond geometry (Å, °)

*D*—H⋯*A*	*D*—H	H⋯*A*	*D*⋯*A*	*D*—H⋯*A*
O1—H1*O*⋯Br	0.82	2.46	3.278 (3)	175
N1—H2*N*⋯Br^i^	0.90 (1)	2.91 (3)	3.696 (4)	147 (5)

Symmetry code: (i) 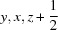.

**Fig. 1.**
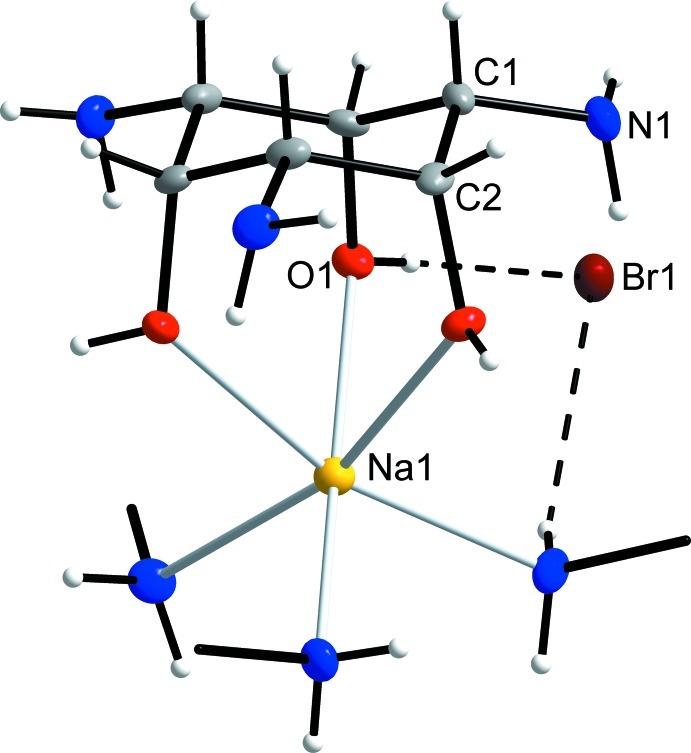
Ellipsoid plot and numbering scheme of a Br anion and an Na(taci) moiety together with the coordinating amino groups of three additional, neighbouring taci units. Displacement parameters are given at the 50% probability level.

**Fig. 2.**
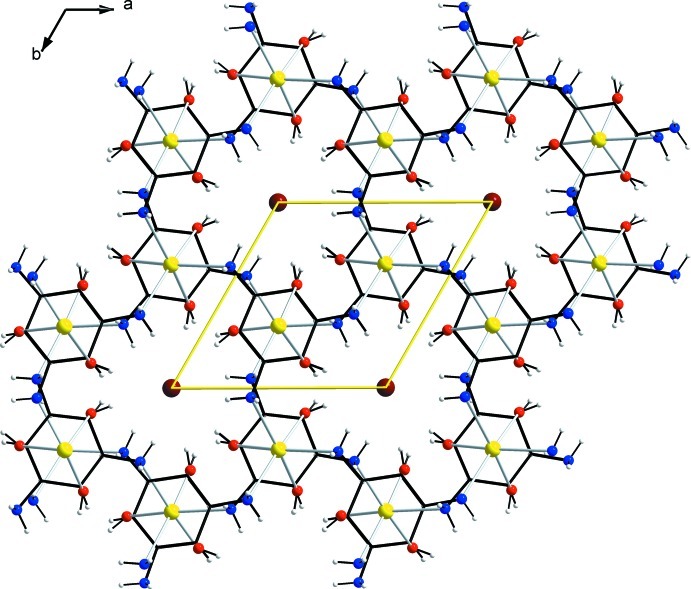
Section of the extended network of the title compound viewed along the c axis.

## supplementary crystallographic information

### Comment

The coordination ability of 1,3,5-triamino-1,3,5-trideoxy-*cis*-inositol (= taci) attracted attention owing to the two distinct chair conformations of this ligand, allowing metal binding either *via* three axial nitrogen or three axial oxygen donors (Hegetschweiler, 1999). For the free taci ligand and its protonation products, a chair conformation with axial hydroxy groups has been asserted (Reiss *et al.*, 1999). However, binding of divalent transition metal cations such as Cu^2+^ usually occurs *via* three axial amino groups (Reiss *et al.*, 1998). The binding of Na^+^ to a protonated bis-taci unit (where two taci-moieties are connected *via* a O—CH_2_—CH_2_—O bridge) occurred *via* the axial hydroxy groups (Bartholomä *et al.*, 2010).

In the title compound, [Na(C_6_H_15_N_3_O_3_)]^+^Br^-^, the cation, anion and the taci ligand have site symmetry 3. The taci ligand also adopts a chair conformation with axial hydroxy groups, which bind to the Na^+^ cation (Na—O distance: 2.409 (4)Å). The puckering parameters (Cremer & Pople, 1975) of the cyclohexane ring are *Q* = 0.531Å, φ = 0.0°, θ = 180.0°. Interlinking of the Na^+^ cation to three equatorial amino groups of three neighbouring taci ligands (Na—N distance: 2.556 (4)Å) generates a distorted octahedral coordination geometry around the sodium ion. Due to symmetry, the three oxygen and the three nitrogen donors form each two parallel, equilateral triangles with a twist angle τ of 56.4°. This value indicates that the *fac*-NaO_3_N_3_ coordination geometry adopts *C*_3v_ symmetry quite closely. The bromide anion is hydrogen-bonded to three hydroxy groups of three adjacent taci ligands. Additionally, three N—H···Br contacts are formed (see Table 1). These N—H···Br contacts may be interpreted as weak hydrogen bonds. The three (N—)H and the three (O—)H hydrogen atoms form again two parallel, equilateral triangles with a twist angle τ = 32.4°. The six hydrogen atoms, which encapsulate the bromide ion, constitute thus a polyhedron which is just an intermediate form between a trigonal prism (τ = 0°) and a trigonal antiprism (τ = 60°). Notably, the bromide ion is not located in the centre of this polyhedron. It is significantly displaced towards the three (O—)H hydrogen atoms. Inspection of the structure further reveals an intermolecular N···O separation of 2.831 (6)Å, a value which falls in a range expected for N—H···O hydrogen bonding. However, the corresponding N—H···O angle of 110 (6)° is very acute and would not be in agreement with such an interpretation.

### Experimental

The title compound was first isolated unintentionally by Egli (1994) in the reaction of K_2_ReBr_6_, NaOCH_3_ and taci. In our study, it was prepared by adding equimolar amounts of NaBr and taci in small portions to boiling MeOH until a saturated solution was obtained. The solution was filtered hot and was allowed to cool slowly to room temperature, yielding colourless single crystals suitable for crystal structure analysis.

### Refinement

All hydrogen atoms were identified in difference syntheses. In the latest stages of refinement, the coordinates of the N– and C– bonded hydrogen atoms were refined, whereas the coordinates of the (O—)H hydrogen atom had to be constrained using the AFIX 83 option of the *SHELXL* program. The N—H bond lengths were restrained to 0.90Å. All *U*_iso_ values, besides those of the CH groups, were refined freely.

### Crystal data

**Table:**

[Na(C_6_H_15_N_3_O_3_)]Br	*D*_x_ = 1.864 Mg m^−^^3^
*M**_r_* = 280.10	Mo *K*α radiation, λ = 0.71073 Å
Trigonal, *P*31*c*	Cell parameters from 99 reflections
Hall symbol: P 3 -2c	θ = 6.6–14.9°
*a* = 8.0491 (10) Å	µ = 4.15 mm^−^^1^
*c* = 8.8953 (18) Å	*T* = 153 K
*V* = 499.10 (13) Å^3^	Block, colorless
*Z* = 2	0.57 × 0.45 × 0.28 mm
*F*(000) = 284	

### Data collection

**Table:**

Siemens P4 diffractometer	682 reflections with *I* > 2σ(*I*)
Radiation source: fine-focus sealed tube	*R*_int_ = 0.077
Graphite monochromator	θ_max_ = 26.5°, θ_min_ = 2.9°
ω scan	*h* = −9→9
Absorption correction: integration (*XPREP*; Bruker, 2008) using indexed faces	*k* = −10→10
*T*_min_ = 0.101, *T*_max_ = 0.331	*l* = −11→11
3954 measured reflections	3 standard reflections every 100 reflections
690 independent reflections	intensity decay: none

### Refinement

**Table:**

Refinement on *F*^2^	Hydrogen site location: inferred from neighbouring sites
Least-squares matrix: full	H atoms treated by a mixture of independent and constrained refinement
*R*[*F*^2^ > 2σ(*F*^2^)] = 0.036	*w* = 1/[σ^2^(*F*_o_^2^) + (0.015*P*)^2^ + 2.*P*] where *P* = (*F*_o_^2^ + 2*F*_c_^2^)/3
*wR*(*F*^2^) = 0.078	(Δ/σ)_max_ < 0.001
*S* = 1.05	Δρ_max_ = 0.42 e Å^−^^3^
690 reflections	Δρ_min_ = −0.55 e Å^−^^3^
59 parameters	Extinction correction: *SHELXL97* (Sheldrick, 2008), Fc^*^=kFc[1+0.001xFc^2^λ^3^/sin(2θ)]^-1/4^
3 restraints	Extinction coefficient: 0.024 (4)
Primary atom site location: structure-invariant direct methods	Absolute structure: Flack (1983), 340 Friedel pairs
Secondary atom site location: difference Fourier map	Flack parameter: −0.03 (3)

### Special details

**Table:**

Geometry. All e.s.d.'s (except the e.s.d. in the dihedral angle between two l.s. planes) are estimated using the full covariance matrix. The cell e.s.d.'s are taken into account individually in the estimation of e.s.d.'s in distances, angles and torsion angles; correlations between e.s.d.'s in cell parameters are only used when they are defined by crystal symmetry. An approximate (isotropic) treatment of cell e.s.d.'s is used for estimating e.s.d.'s involving l.s. planes.
Refinement. Refinement of *F*^2^ against ALL reflections. The weighted *R*-factor *wR* and goodness of fit *S* are based on *F*^2^, conventional *R*-factors *R* are based on *F*, with *F* set to zero for negative *F*^2^. The threshold expression of *F*^2^ > σ(*F*^2^) is used only for calculating *R*-factors(gt) *etc*. and is not relevant to the choice of reflections for refinement. *R*-factors based on *F*^2^ are statistically about twice as large as those based on *F*, and *R*- factors based on ALL data will be even larger.

### Fractional atomic coordinates and isotropic or equivalent isotropic displacement parameters (Å^2^)

**Table:**

	*x*	*y*	*z*	*U*_iso_*/*U*_eq_	
Na	0.3333	0.6667	0.1288 (3)	0.0163 (6)	
Br	1.0000	1.0000	0.22830 (19)	0.0196 (3)	
N1	0.6544 (6)	0.6032 (6)	0.4871 (4)	0.0180 (8)	
H1N	0.638 (9)	0.569 (8)	0.390 (2)	0.027 (15)*	
H2N	0.768 (4)	0.707 (5)	0.508 (7)	0.024 (14)*	
C1	0.4981 (6)	0.6393 (6)	0.5290 (5)	0.0141 (9)	
H1	0.494 (8)	0.637 (8)	0.627 (5)	0.017*	
C2	0.5288 (6)	0.8341 (6)	0.4803 (5)	0.0144 (9)	
H2	0.638 (8)	0.926 (8)	0.526 (6)	0.017*	
O1	0.5558 (4)	0.8645 (5)	0.3206 (3)	0.0155 (6)	
H1O	0.6689	0.9049	0.2998	0.023 (15)*	

### Atomic displacement parameters (Å^2^)

**Table:**

	*U*^11^	*U*^22^	*U*^33^	*U*^12^	*U*^13^	*U*^23^
Na	0.0170 (9)	0.0170 (9)	0.0148 (13)	0.0085 (5)	0.000	0.000
Br	0.0178 (3)	0.0178 (3)	0.0232 (4)	0.00892 (14)	0.000	0.000
N1	0.0150 (18)	0.023 (2)	0.0201 (19)	0.0123 (16)	−0.0004 (16)	0.0018 (17)
C1	0.016 (2)	0.021 (2)	0.0103 (19)	0.0130 (19)	0.0007 (17)	0.0021 (18)
C2	0.016 (2)	0.015 (2)	0.0085 (18)	0.0055 (18)	−0.0035 (17)	−0.0003 (17)
O1	0.0137 (16)	0.0197 (16)	0.0122 (13)	0.0076 (14)	0.0021 (13)	0.0031 (12)

### Geometric parameters (Å, º)

**Table:**

Na—O1^i^	2.409 (4)	N1—H2N	0.900 (10)
Na—O1	2.409 (4)	C1—C2	1.523 (6)
Na—O1^ii^	2.409 (4)	C1—C2^ii^	1.526 (6)
Na—N1^iii^	2.556 (4)	C1—H1	0.87 (5)
Na—N1^iv^	2.556 (4)	C2—O1	1.440 (5)
Na—N1^v^	2.556 (4)	C2—C1^i^	1.526 (6)
N1—C1	1.473 (5)	C2—H2	0.91 (6)
N1—Na^vi^	2.556 (4)	O1—H1O	0.8200
N1—H1N	0.895 (10)		
			
O1^i^—Na—O1	75.37 (13)	C1—N1—H2N	110 (4)
O1^i^—Na—O1^ii^	75.37 (13)	Na^vi^—N1—H2N	103 (4)
O1—Na—O1^ii^	75.37 (13)	H1N—N1—H2N	114 (5)
O1^i^—Na—N1^iii^	94.37 (12)	N1—C1—C2	114.6 (4)
O1—Na—N1^iii^	164.19 (15)	N1—C1—C2^ii^	110.1 (4)
O1^ii^—Na—N1^iii^	90.53 (12)	C2—C1—C2^ii^	113.7 (4)
O1^i^—Na—N1^iv^	90.53 (12)	N1—C1—H1	106 (4)
O1—Na—N1^iv^	94.37 (12)	C2—C1—H1	107 (4)
O1^ii^—Na—N1^iv^	164.19 (15)	C2^ii^—C1—H1	105 (4)
N1^iii^—Na—N1^iv^	97.78 (14)	O1—C2—C1	112.9 (3)
O1^i^—Na—N1^v^	164.19 (15)	O1—C2—C1^i^	109.1 (4)
O1—Na—N1^v^	90.53 (12)	C1—C2—C1^i^	110.8 (4)
O1^ii^—Na—N1^v^	94.37 (12)	O1—C2—H2	107 (3)
N1^iii^—Na—N1^v^	97.78 (14)	C1—C2—H2	108 (4)
N1^iv^—Na—N1^v^	97.78 (14)	C1^i^—C2—H2	109 (3)
C1—N1—Na^vi^	116.3 (3)	C2—O1—Na	126.0 (3)
C1—N1—H1N	107 (4)	C2—O1—H1O	109.5
Na^vi^—N1—H1N	106 (4)	Na—O1—H1O	114.4
			
Na^vi^—N1—C1—C2	162.1 (3)	C1^i^—C2—O1—Na	−65.3 (4)
Na^vi^—N1—C1—C2^ii^	−68.3 (4)	O1^i^—Na—O1—C2	41.5 (3)
N1—C1—C2—O1	57.6 (5)	O1^ii^—Na—O1—C2	−36.9 (3)
C2^ii^—C1—C2—O1	−70.2 (5)	N1^iii^—Na—O1—C2	−9.3 (6)
N1—C1—C2—C1^i^	−179.8 (3)	N1^iv^—Na—O1—C2	130.9 (3)
C2^ii^—C1—C2—C1^i^	52.5 (6)	N1^v^—Na—O1—C2	−131.3 (3)
C1—C2—O1—Na	58.3 (5)		

Symmetry codes: (i) −*y*+1, *x*−*y*+1, *z*; (ii) −*x*+*y*, −*x*+1, *z*; (iii) *x*−*y*, −*y*+1, *z*−1/2; (iv) −*x*+1, −*x*+*y*+1, *z*−1/2; (v) *y*, *x*, *z*−1/2; (vi) *y*, *x*, *z*+1/2.

### Hydrogen-bond geometry (Å, º)

**Table:**

*D*—H···*A*	*D*—H	H···*A*	*D*···*A*	*D*—H···*A*
O1—H1*O*···Br	0.82	2.46	3.278 (3)	175
N1—H2*N*···Br^vi^	0.90 (1)	2.91 (3)	3.696 (4)	147 (5)

Symmetry code: (vi) *y*, *x*, *z*+1/2.
